# Postpartum Pyomyoma, a Rare Complication of Sepsis Associated with Chorioamnionitis and Massive Postpartum Haemorrhage Treated with an Intrauterine Balloon

**DOI:** 10.1155/2015/609205

**Published:** 2015-06-14

**Authors:** Mandeep Kaler, Ruth Gailer, Joseph Iskaros, Anna L. David

**Affiliations:** Institute for Women's Health, University College London Hospitals NHS Foundation Trust, 235 Euston Road, London WC1E 6AU, UK

## Abstract

We report the successful treatment of a postpartum pyomyoma, a rare but serious complication of uterine leiomyomata in a 28-year-old primigravida. The patient was treated for an *Escherichia Coli* (*E. Coli*) urinary tract infection (UTI) at 16 weeks of gestation. She had asymptomatic short cervical length on ultrasound scan at 20 weeks that was managed conservatively due to the presence of further UTI and received antibiotics. She was known to have a left sided intramural leiomyoma. She presented with abdominal pain and vaginal bleeding at 23^+1^ weeks of gestation and the next day she had spontaneous vaginal delivery and collapsed with *E. Coli* septic shock, massive postpartum haemorrhage, and disseminated intravascular coagulation and was successfully treated with oxytocic drugs, a Rusch intrauterine balloon, and intravenous antibiotics. Eleven days postnatally she re-presented with systemic sepsis and was treated for retained products of conception. Sepsis persisted and investigations showed a postpartum pyomyoma that was initially managed with intravenous antibiotics to avoid surgery. Ultimately she required laparotomy, drainage of pyomyoma, and myomectomy. Postoperative recovery was good and the patient had a successful pregnancy two years later.

## 1. Introduction

Leiomyomas (fibroids) are benign smooth muscle tumours of the uterus affecting up to three-quarters of all women [1–4]. Risk factors associated with an increased incidence include obesity, nulliparity, family history, Afro-Caribbean heritage, and early menarche [4–6].

A pyomyoma is a rare but serious complication of uterine leiomyomata that usually arises secondary to infarction followed by infection of the degenerating fibroid. They are commonly seen in the postpartum or postmenopausal period [1, 4]. Of all the cases reported in the literature since 1945, eighteen have been pregnancy related, half of which resulted in hysterectomy Table 1. The mortality associated with pyomyoma is 21% with no deaths reported in those associated with pregnancy [1–3]. With the increased use of interventions to conserve the uterus rather than immediate recourse to hysterectomy in controlling postpartum haemorrhage, pyomyoma may become more frequently encountered in clinical practice in women known to have uterine leiomyomata. Postpartum haemorrhage is common after chorioamnionitis and the devices such as intrauterine balloons in the presence of infection may increase the chance of pyomyoma.

Management during pregnancy is complicated by the desire to avoid hysterectomy and to preserve future fertility, particularly when the pyomyoma is associated with late miscarriage. We present a case of postpartum pyomyoma with initial conservative management in which later myomectomy was successful with conservation of the uterus and a subsequent live birth.

## 2. Case

A 28-year-old Caucasian primigravida, presented with lower abdominal pain and a small amount of vaginal bleeding at 23^+1^ weeks of gestation. She was known to have two homogeneous subserous fibroids on the left lateral anterior and left lateral wall of the uterus [35 × 39 × 50 mm and 49 × 81 × 100 mm, resp.], seen first on a dating ultrasound scan. The patient was heterozygous for methyl-tetrahydrofolate reductase [MTHFR], had an increased factor VIII level, and was taking aspirin 75 mg daily. There was no history of cervical surgery. Previous vaginal swabs had confirmed that she was a carrier of Group B* Streptococcus* [GBS]. She had received antibiotic treatment abroad for a urinary tract infection [UTI] at 16-week gestation. At routine anomaly scan, the cervical length measured 4 mm and the risk of preterm labour was discussed; she had no symptoms of threatened preterm labour. She was counselled that the short cervix was likely to be related to the presence of earlier UTI, and a cerclage was not recommended. Furthermore a midstream urine sample showed a second UTI (cephalosporinase-producing* E. coli*), for which the patient was treated with a course of nitrofurantoin to which the bacteria were sensitive.

On admission at 23^+1^ weeks, speculum examination confirmed a diagnosis of preterm prelabour rupture of membranes. The patient was prescribed steroids for fetal lung maturation and oral erythromycin to prevent infection. Intravenous [IV] antibiotics were commenced in accordance with protocol due to the positive GBS status. Serum blood tests subsequently showed a mildly raised white cell count [11.18 × 10^9^/L] and elevated inflammatory markers [CRP 95.5 mg/L]. Expectant management was discussed and preferred by the patient at this point. She remained apyrexial but developed a persistent tachycardia [110–120 bpm]. The following day, her CRP rose [151.3 mg/L], and she developed a macular-papular rash over her trunk, upper limbs, and abdomen suggesting systemic infection. Vaginal examination confirmed that she was in early labour. Less than four hours later, a live born female infant was delivered vaginally as a footling breech in membranes [birth weight 560 g]. The placenta was delivered twenty-nine minutes later and was noted to be fragmented. Following delivery there was a maternal collapse, with an associated massive postpartum haemorrhage [PPH, 1750 mL] secondary to uterine atony and sepsis with suspected disseminated intravascular coagulopathy [DIC]. The patient was immediately transferred to theatre, where she became hypotensive and pyrexial. She was given a general anaesthetic and standard major obstetric haemorrhage management was commenced to include resuscitation with IV fluids, blood, fresh frozen plasma, platelets, and uterotonics. The remaining fragments of placenta were evacuated manually and the cavity was checked with a transabdominal ultrasound and was found to be empty. A Rusch balloon was placed via the vagina into the uterus because of continued uterine atony and PPH, which then resolved within the hour. She was transferred to the Intensive Care Unit for further care and was commenced on IV meropenem, Teicoplanin, and metronidazole following microbiology advice.

She was extubated the next day and the intrauterine balloon was removed uneventfully. Seven days of IV antibiotic therapy was given with good response both clinically and in her markers of infection. Genital swabs and placental tissue at this time grew the same cephalosporinase producing* E. coli* confirming chorioamnionitis. She was discharged eight days after delivery. Unfortunately, the baby died on the neonatal unit during this time.

Three days following discharge, the patient presented to hospital with fever, lethargy, and abdominal discomfort. The uterus was tender on palpation and her inflammatory markers were elevated again. She was commenced on IV Co-Amoxiclav for presumed ongoing sepsis. An ultrasound showed a thickened endometrium, the previously documented fibroids (of similar dimensions), and apparent retained products of conception measuring 19 × 33 × 46 mm. Accordingly, she underwent evacuation of retained products of conception [ERPC] the following day under ultrasound guidance. At the operation a significant amount of products of conception were removed and confirmed on histological examination. The uterus was shown to be empty on ultrasound examination.

Following the ERPC the patient continued to spike fevers and reported no significant improvement in her symptoms. Blood and urine cultures continued to yield no growth. As a result IV Teicoplanin was added to her antibiotic regimen on microbiology advice and a CT scan of the Abdomen and Pelvis with contrast was performed three days after the ERPC in search of another intra-abdominal source of sepsis. A multiple thick-walled low attenuation cystic adnexal mass lesion was seen, particularly marked on the left [11 × 7 × 7 mm] of similar dimensions to the previously seen larger fibroid. A differential diagnosis of complex ovarian cyst or tuboovarian abscess was proposed, but a repeat transvaginal ultrasound showed normal ovaries, no evidence of a pelvic collection, and the fibroids were noted to be unchanged from the previous ultrasound scan. A diagnosis of a degenerating pyomyoma was made. Myomectomy was discussed but due to the inherent risk of hysterectomy and the desire to preserve her uterus for future fertility, both the team and the patient were keen to avoid surgical intervention unless significant clinical deterioration occurred.

**Figure 1:**
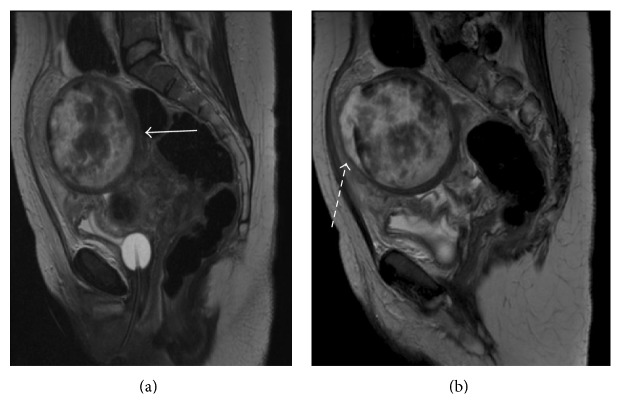
MRI of the PELVIS. (a) Necrotic 8.2 cm subserosal fibroid [arrow]. (b) Necrotic 8.2 cm subserosal fibroid with an encapsulated collection lying anterior to the fibroid suggesting rupture [dashed arrow].

Over the next four days the swinging pyrexia continued, associated with another peak in serum markers of inflammation. An MRI of the Abdomen and Pelvis, performed at 22 days postpartum, confirmed the working diagnosis of pyomyoma, revealing an 8.2 cm subserosal fibroid, related to the fundus with necrotic appearances and possible encapsulated collection lying anterior to the fibroid suggesting rupture [Figures 1(a) and 1(b)]. The patient had not responded clinically to conservative management. Therefore, at thirty days postpartum the patient underwent a planned laparoscopy to evaluate the abdominal cavity. The laparoscopy confirmed a fibroid adherent to the abdominal wall with omental wrapping and laparotomy was performed via Pfannenstiel incision. The pyomyoma was drained and a myomectomy was performed. The other smaller fibroid was not removed as its appearance was vascular and nonnecrotic, and removal represented a significant risk of bleeding. Postoperatively the patient recovered well and was discharged five days later with oral antibiotics. Histological examination of the pyomyoma confirmed extensive infarction and cystic degeneration. She conceived naturally two years later and delivered a live healthy baby.

## 3. Discussion

A pyomyoma is a rare and serious condition, which, as our case illustrates, is often difficult to diagnose. Myomas are thought to become infected as a result of bacterial colonization following infarction. Organisms, such as* Clostridium* spp.,* Staphylococcus aureus*,* Streptococcus milleri*,* Proteus* sp.,* Enterococcus faecalis*,* Actinomyces meyeri*,* Serratia marcescens*,* Klebsiella pneumoniae*, and* Streptococcus agalactiae*, have been recognised as causative bacteria resulting in pyomyoma (Table 1) [4, 6].

Pyomyoma can develop through different routes of spread: direct invasion from the uterine cavity, spread from adjacent structures [e.g., bowel], and lymphatic or haematogenous spread [4, 6, 7]. Most commonly the patient presents with complaints of fever, abdominal pain, or an abdominal mass, potentially leading to fatal complications including ruptured pyomyoma, peritonitis, and septic shock [4, 7]. In our case, the source of infection is likely to have occurred secondary to chorioamnionitis and systemic sepsis and possibly directly via the uterine cavity during ERPC. The chorioamnionitis is likely to have developed secondary to the two UTIs and associated short cervical length. A cervical cerclage was not recommended when the short cervical length was identified due to the diagnosis of a UTI and the likelihood that cerclage would fail or even be detrimental to the patient in the presence of infection. There were no other causes of short cervical length in this patient (cervical surgery such as cone biopsy or uterine anomaly) and the patient was carefully counselled about the risks of preterm labour when a short cervical length was identified at the routine anomaly ultrasound scan.

The uterine cavity was checked to be empty by ultrasound examination prior to placement of the balloon but it is not unusual for products of conception to remain after a manual removal of placenta is required. The uterine balloon used for management of uterine atony and PPH represented a significant source of infection, particularly since it was placed at the time of overwhelming sepsis. The decision to place such a balloon is not taken lightly but fortunately was sufficient to manage the immediate PPH. The next steps to manage PPH if the balloon had been unsuccessful would have been uterine artery embolization, with its associated risks of uterine infarction and subfertility or finally hysterectomy.

Being such a rare condition, clinicians face the difficulty in making such a diagnosis early, as differential diagnoses such as tuboovarian abscess, pyometra, malignancy, and perforated viscus must be considered. The size and site of the fibroid do not appear to be related to the development of pyomyoma in this or other cases reported (Table 1). Onset of symptoms may be sudden or insidious in nature and as our case illustrates, postpartum pyomyoma may present over days to weeks after delivery. In addition, despite initial improvement in symptoms of infection with IV antibiotics, the patient's clinical status deteriorated over a total of 3 weeks, suggesting that a continuing infective process was present.

This patient was investigated with a variety of imaging modalities to aid diagnosis. We found that ultrasound was able to determine the size and position of the fibroid but did not prove so useful for diagnosis of pyomyoma. CT pelvis and MRI were more useful, and characteristically those of pyomyoma showed large heterogeneous echogenic pelvic mass with a solid and cystic component. A ruptured pyomyoma can be identified by air and debris within the fibroid, discontinuity of the fibroid wall, intraperitoneal free air, and ascites [5, 7]. In the MRI of our patient some of these features were seen (solid and cystic component and discontinuity of fibroid wall) and were suggestive of pyomyoma rupture.

Most of the reported cases in the literature show that definitive management requires myomectomy or hysterectomy and IV antibiotics. There have been recent reports of the use of CT guided drainage with a pigtail catheter as a first-line treatment option of a pyomyoma. This method of treatment is desirable especially when trying to preserve fertility. However, if unsuccessful the patient would still require definitive surgical treatment [8].

Despite the rare nature of this pregnancy complication, a pyomyoma should be considered early if a patient presents with a history of a fibroid uterus and use of interventions such as a uterine balloon, with signs and symptoms of sepsis and without an alternative primary focus of infection. Use of IV antibiotics initially may appear to improve symptoms, but ultimately surgical treatment, with preservation of the uterus for future fertility, is necessary.

## Figures and Tables

**Table 1:** Details of reports of pyomyoma in association with pregnancy.

Author	Age (years)	Gestation	Background	Presentation	Organism	Treatment	Complications	Outcome
^*∗*^Present case	28	23 + 1 weeks	Known leiomyoma Preterm labour GBS positive	Abdominal pain PV bleeding Fever	*Escherichia coli *	Antibiotics Laparotomy and myomectomy	Septicaemia	Well

Sirha et al. [9]	37	10 days postpartum	27-week preterm delivery Caesarean section	Fever Vomiting	Unknown	Antibiotics Total abdominal hysterectomy	Sepsis	Well

Del Borgo et al. [10]	37	30 days postpartum	SVD at 39 weeks	Fever	*Sphingomonas paucimobilis *	Antibiotics Abdominal myomectomy	Septicaemia	Well

Kobayashi et al. [11]	28	20 weeks	Degenerating leiomyoma at 18-week gestation	Abdominal pain Fever Vaginal discharge	Anaerobic Gram-negative rods	Exploratory laparotomy Myomectomy at 21 weeks of gestation	Peritonitis	Well Caesarean section at 37 weeks

Shaaban et al. [12]	30	8 weeks postpartum	Caesarean section Known leiomyoma	Fever Abdominal pain	*Staphylococcus lugdunensis *	Antibiotics Exploratory laparotomy Myomectomy	Peritonitis	Well

Laubach et al. [8]	31	29 weeks	Preterm prelabour rupture of membranes Footling breech Caesarean section Previous myomectomy	Fever	*Enterococcus faecalis Streptococcus* species	Antibiotics CT guided drainage Subtotal abdominal hysterectomy	Wound infection	Well

Laubach et al. [8]	35	33 weeks	Preterm labour Footling breech Chorioamnionitis Caesarean section Previous myomectomy	Fever Abdominal pain	*Escherichia coli Candida albicans * *Candida dubliniensis *	Antibiotics CT guided drainage	N/A	Well

Laubach et al. [8]	31	18 hours after ERPC for miscarriage	13-week pregnancy	Fever Abdominal pain	*Escherichia coli *	Antibiotics CT guided drainage	N/A	Well

Nguyen and Gruenewald [13]	40	3 weeks postpartum	Caesarean section at 41 weeks Chorioamnionitis	Fever Abdominal pain	*Escherichia coli *	Antibiotics Total abdominal hysterectomy	Sepsis	Well

Mason et al. [4]	29	21 days postpartum	SVD at term MROP	Fever Abdominal pain Vomiting	No growth	Antibiotics Exploratory laparotomy Myomectomy	Sepsis Endometritis	

Karcaaltincaba and Sudakoff [7]	36	7 days after spontaneous 2nd trimester miscarriage	17-week gestation Known leiomyoma	Abdominal pain Fever	*Peptostreptococcus tetradrus *	Exploratory laparotomy Myomectomy	Peritonitis	Well

Lin et al. [14]	33	6 days postpartum	33 weeks Preterm labour Caesarean section Known leiomyoma	Abdominal pain Fever	*Candida parapsilosis *	Antibiotics Total abdominal hysterectomy	Septic shock Endometritis Wound infection	Well

Grüne et al. [15]	44	26 weeks	Known leiomyoma Caesarean section	Fever	*Klebsiella pneumoniae *	Antibiotics Myomectomy	Sepsis	Well

Prahlow et al. [16]	31	12 weeks	Ongoing pregnancy Intravenous drug abuse Previous PID	Abdominal pain Constipation	*Staphylococcus aureus *	Total abdominal hysterectomy & BSO Antibiotics	Peritonitis	Well

Tobias et al. [17]	32	10 weeks after surgical TOP at 15 weeks	Known leiomyoma	Abdominal pain Fever	*Enterococcus faecalis *	Antibiotics Total abdominal hysterectomy & BSO	Peritonitis	Well

Prichard et al. [18]	37	9 weeks after 2nd trimester miscarriage	Known leiomyoma Exploratory Laparotomy 18 weeks earlier for abdominal mass	Fever Weight loss	*Streptococcus milleri *	Antibiotics Total abdominal hysterectomy & BSO	Infective endocarditis	Well

Wong et al. [19]	29	18 weeks	Intrauterine device in situ Chorioamnionitis leading to miscarriage	Fever	*Staphylococcus aureus Serratia marcescens *	Antibiotics Total abdominal hysterectomy & LSO	Hemoperitoneum	Well

Ruch [20]	32	24 weeks	Preterm labour Known leiomyoma	Fever Abdominal pain Lethargy	Gram-positive cocci	Antibiotics Total abdominal hysterectomy & BSO	Peritonitis	Well

Dubois and Neumann [21]	29	3 weeks postpartum	SVD at term	Fever Abdominal pain	Unknown	Antibiotics Vaginal expulsion/myomectomy	N/A	Well

^*∗*^Case described in this paper.

BSO: bilateral salpingo-oophorectomy; NVD: normal vaginal delivery; TOP: termination of pregnancy; MROP: manual removal of placenta; ERPC: evacuation of retained products of conception; PID: pelvic inflammatory disease.
